# Charles West on Children

**Published:** 1874-09

**Authors:** 


Lectures on the Diseases of Infancy and Childhood. By Charles West, M.D., Fellow of the Royal College of Physicians; Physician to the Hospital for Sick Children. Fifth American, from the sixth revised and enlarged English Edition. Philadelphia: Henry C. Lea, 1874. Octavo, leather, pp. 678. Price, $5.50; cloth, $4.50.

The dignity, the conservatism, the rare good sense of Dr. Charles West, of London, give importance and weight to his utterances upon any medical subject, but more especially upon the diseases of women and of children, to the study of which he has devoted a lifetime. The spirit, the animus of the author are so beautifully foreshadowed in his preface we are tempted to extract the following as a fitting commentary upon his work:

"Any one in his fifty-eighth year must feel it very doubtful whether his life will be so prolonged as to allow him to take part in the publication of another edition of a book which first appeared more than twenty-five years ago; or whether some better, completer work will not before long occupy its place. But be this as it may, to me it is no small satisfaction to feel that future laborers will have their tasks lightened by my endeavors; that while thirty years ago there was not a single hospital for children in the whole British dominions or in America, the Hospital for sick Children in Great Ormond Street, and its thirty daughters, will now tell, if my name should be at all remembered, that it was permitted me to live not altogether to myself, but in some small degree at least to serve my generation, and to help those little ones whom I so much love."

Dr. West's work has been carefully revised and brought up to date; it is sufficiently progressive for the times, and yet has lost nothing of the solidity and conservatism so characteristic of its author. It will be found a safe and excellent text-book for the student, and a useful guide to the practitioner.

---

Annual Announcements.—Vanderbilt University, Nashville, Tennessee; Washington University, Baltimore, Maryland; University of Michigan, Ann Arbor; American Social Science Association.

